# THE RELATIONSHIP BETWEEN LONG-TERM CARE OMBUDSMAN PROGRAM SPENDING AND NURSING HOME OUTCOMES

**DOI:** 10.1093/geroni/igad104.2090

**Published:** 2023-12-21

**Authors:** Katherine Kennedy, Cyrus Kosar, Kali Thomas

**Affiliations:** Providence VA Medical Center, Providence, Rhode Island, United States; Brown University, Providence, Rhode Island, United States; Johns Hopkins University, Baltimore, Maryland, United States

## Abstract

As part of the Older Americans Act, State Long-term Care Ombudsman Programs (LTCOP) serve older adults in nursing homes (NHs) and board and care facilities. The National Academies of Science, Engineering, and Medicine called for increased LTCOP funding to improve NH quality. We hypothesized that increases in spending on LTCOP would be associated with a lower prevalence of NH residents with low-care needs who could be cared for in the community and fewer residents taking antipsychotics. We conducted a secondary analysis using the National Ombudsman Reporting System, NH facility-level data from LTCfocUS.org for 15,592 US NHs (2011-2018), and the Area Health Resource File. Using a two-way fixed effects model with standard errors clustered at the facility-level, we examined the relationship between LTCOP spending per LTC bed at the state-level and outcomes, controlling for year, state, facility, and market characteristics. Increased LTCOP spending was associated with a lower proportion of residents in NHs with low-care needs and a lower percent of residents receiving antipsychotics (p < .0001). In 2011, the average share of low-care NH residents (weighted by bed size) was 13.34%, and the share of residents on antipsychotics was 25.95%. For every $25 annual change in LTCOP funds, there was a .84 percentage point decrease in the prevalence of low-care residents for the average NH, and a .80 percentage point decrease in the share of residents receiving antipsychotics. States that have increased funding for their LTCOP observe better outcomes. This documents the need to increase funding to support LTCOP.

